# Human Tumour Immune Evasion via TGF-β Blocks NK Cell Activation but Not Survival Allowing Therapeutic Restoration of Anti-Tumour Activity

**DOI:** 10.1371/journal.pone.0022842

**Published:** 2011-09-06

**Authors:** Erica B. Wilson, Jehan J. El-Jawhari, Abbie L. Neilson, Geoffrey D. Hall, Alan A. Melcher, Josephine L. Meade, Graham P. Cook

**Affiliations:** Leeds Institute of Molecular Medicine, Wellcome Brenner Building, St. James's University Hospital, University of Leeds, Leeds, United Kingdom; Centre de Recherche Public de la Santé (CRP-Santé), Luxembourg

## Abstract

Immune evasion is now recognized as a key feature of cancer progression. In animal models, the activity of cytotoxic lymphocytes is suppressed in the tumour microenvironment by the immunosuppressive cytokine, Transforming Growth Factor (TGF)-β. Release from TGF-β-mediated inhibition restores anti-tumour immunity, suggesting a therapeutic strategy for human cancer. We demonstrate that human natural killer (NK) cells are inhibited in a TGF-β dependent manner following chronic contact-dependent interactions with tumour cells *in vitro*. *In vivo*, NK cell inhibition was localised to the human tumour microenvironment and primary ovarian tumours conferred TGF-β dependent inhibition upon autologous NK cells *ex vivo*. TGF-β antagonized the interleukin (IL)-15 induced proliferation and gene expression associated with NK cell activation, inhibiting the expression of both NK cell activation receptor molecules and components of the cytotoxic apparatus. Interleukin-15 also promotes NK cell survival and IL-15 excluded the pro-apoptotic transcription factor FOXO3 from the nucleus. However, this IL-15 mediated pathway was unaffected by TGF-β treatment, allowing NK cell survival. This suggested that NK cells in the tumour microenvironment might have their activity restored by TGF-β blockade and both anti-TGF-β antibodies and a small molecule inhibitor of TGF-β signalling restored the effector function of NK cells inhibited by autologous tumour cells. Thus, TGF-β blunts NK cell activation within the human tumour microenvironment but this evasion mechanism can be therapeutically targeted, boosting anti-tumour immunity.

## Introduction

Human tumour cells can be recognized and eliminated by the immune system but this selective pressure favours the outgrowth of tumours that have successfully evaded immunity [1]. Indeed, the ability to evade the immune response is now recognized as a hallmark of cancer [2]. Numerous mechanisms of tumour immune evasion have been described [1], [2], [3], [4]. In the case of cytotoxic lymphocytes, loss of MHC class I expression by the tumour prevents cytotoxic T lymphocyte (CTL) recognition. However, loss of MHC class I expression favours natural killer (NK) cell activation. Activated NK cells exocytose their cytotoxic granules leading to the release of pro-apoptotic components (granzymes and perforin) that kill the tumour. In addition, secretion of interferon (IFN)-γ promotes adaptive cellular immunity [5]. Like CTL, NK cells play an important role in tumour immune surveillance but, not surprisingly, their powerful effector functions are countered by evasion strategies that contribute to tumour escape [5], [6], [7], [8]. Human NK cells express several activation receptors important in tumour cell recognition including NKG2D, DNAM-1 and the natural cytotoxicity receptors NKp30, NKp44 and NKp46 and several studies have identified the reduced expression of these molecules and weakened NK cell activity in human cancer [5], [6], [9], [10], [11], [12], [13].

The immunosuppressive cytokine Transforming Growth Factor (TGF)-β plays a key role in tumour immune evasion [14]. In the healthy immune system, TGF-β serves to blunt immune responses, minimising self-reactivity [14], [15]. Tumours exploit this activity, and many other functions of TGF-β, to promote their progression [14], [16]. The levels of TGF-β are often elevated in the serum of cancer patients and this is associated with systemic inhibition of immune function, including weakened NK cell responses, and is associated with a poor prognosis [16], [17], [18]. However, such systemic effects are likely to be limited to patients with a large tumour burden. In contrast, localised immune evasion is likely to operate throughout tumour progression.

There is increasing evidence that the tumour microenvironment plays a key role in regulating tumour growth, angiogenesis and immune surveillance [3], [14]. Tumour antigen-specific CTL and NK cells demonstrate effective anti-tumour activity *in vitro* and infiltrate solid tumours *in vivo*, yet their activity is weakened within the tumour microenvironment [13], [14], [19]. Evidence from animal studies indicates that antagonism of TGF-β activity can restore productive tumour immunity, suggesting a therapeutic strategy [14], [20]. However, the human tumour microenvironment is much less accessible than the peripheral circulation and does not easily lend itself to mechanistic studies or assessment of therapeutic strategies. We have studied human NK cell evasion using model systems and by analysis of the malignant ascites of human ovarian cancer. In this disease, tumour-associated NK cells and tumour cells are relatively easy to access, enabling *in vivo* and *ex vivo* analysis of immune evasion to be performed. Our results indicate a pivotal role for TGF-β in the tumour localised evasion of human NK cells and demonstrate that the suppressive effects of this cytokine on NK cell effector function can be reversed using TGF-β antagonists in wholly autologous, patient-derived systems *ex vivo*.

## Results

### Modelling chronic interactions between human NK cells and tumours

*In vivo*, the interactions between NK cells and tumours are chronic in nature and tumours proliferate and progress in the continual presence of immune effector cells. We modelled this *in vitro* by establishing longer-term co-cultures (one to seven days) between tumour cell lines and interleukin (IL)-15 stimulated NK cells from the peripheral blood of healthy donors. The NK cells from these co-cultures exhibited reduced cell surface expression of the activation receptors NKp30, NKG2D and DNAM-1, whereas expression of NKp46 was largely unaffected (Figure S1). The alterations in NK cell surface phenotype were accompanied by decreased IFN-γ production and reduced cytotoxic granule exocytosis following restimulation of the NK cells with tumour targets (Figure S1). However, IFN-γ production after stimulation with PMA and ionomycin was unaffected by prior co-culture, suggesting that the inhibition of effector function was most likely due to reduced expression of activating receptors rather than inhibition of downstream signalling pathways (Figure S1). The inhibition of NK cells by tumours was reversible, required NK-tumour cell contact and was exerted by several tumour cell types. Furthermore, a comparison of malignant versus immortalised keratinocytes revealed greater inhibition by the cancer cells, suggestive of a tumour immune evasion mechanism (Figure S1).

### Chronic inhibition of NK cells is mediated by TGF-β

**Figure 1:**
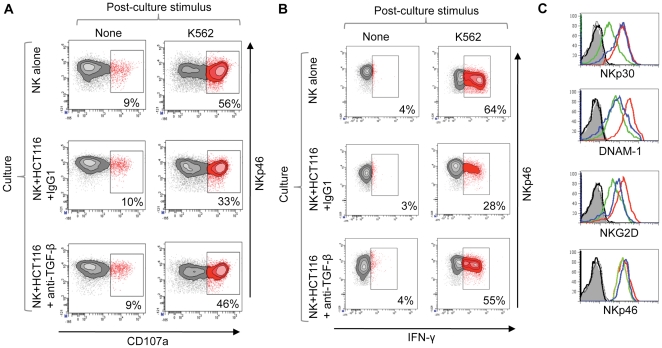
TGF-β dependent inhibition of NK cells following chronic interaction with tumour cells. (A and B) NK cell effector function was analysed following 48 hr interaction with the colorectal cancer cell line HCT116. NK cells were cultured in the presence of IL-15, either with or without HCT116 cells, and in the presence of an anti-TGF-β antibody (or a control antibody) as indicated. Granule exocytosis (A) and IFN-γ production (B) were then analysed following restimulation with K562. The percentage of responding cells for each treatment is indicated. Both assays are one of two independent experiments. Killing of K562 cells was also inhibited in a TGF-β dependent manner (Figure S2). (C) NK cell activation receptor expression (as indicated) was assayed after co-culture with HCT116 in the presence of anti-TGF-β antibody (blue histogram), a control antibody (green histogram) or by NK cells cultured in the absence of tumour cells (red histogram). Isotype control stains are shown in grey and black.

The pattern of inhibition of NK cell surface receptor expression mediated by tumour cells closely resembled that observed when IL-15 stimulated NK cells were treated with the immunosuppressive cytokine TGF-β [21], [22], [23]. Inclusion of an anti-TGF-β antibody into the co-culture between IL-15 stimulated NK cells and tumour cells revealed that TGF-β blockade restored NK cell effector function (Figure 1A, B and Figure S2) and that this was associated with a restoration of NKp30 expression at the cell surface and increases in both DNAM-1 and NKG2D molecules (Figure 1C). Thus, chronic interactions between tumour and NK cells resulted in the TGF-β dependent inhibition of NK cell effector function via the reduced expression of NK cell activation receptors.

### TGF-β antagonises IL-15 induced expression of genes encoding NK cell activation receptors and components of the cytotoxic apparatus

**Figure 2:**
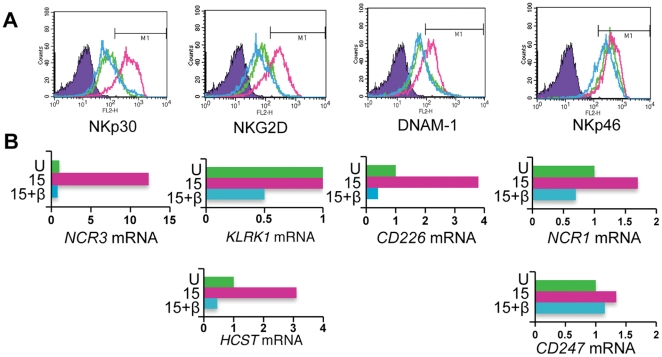
TGF-β antagonises IL-15 induced gene expression of NK cell activation receptors. (A) Cell surface expression of NK cell activation receptors (as indicated) by unstimulated NK cells (green histogram), IL-15 stimulated NK cells (red histogram) or NK cells treated with IL-15 and TGF-β (blue histogram). Cells were cultured for 48 hrs. Isotype control stains are shown in purple. (B) Receptor gene expression in cultures of unstimulated NK cells (U), IL-15 stimulated cells (15) and IL-15 plus TGF-β treated NK cells (15+β). Steady-state mRNA levels were determined by quantitative (q)RT-PCR and the results expressed as arbitrary units (with expression in unstimulated NK cells defined as 1 unit; note the different scales). The colours correspond to the flow cytometry plots shown in (A).

We then analysed the mechanisms by which TGF-β inhibits NK cell function. TGF-β exerts its effects largely via the SMAD signalling pathway and the regulation of gene expression [22], [24], [25]; TGF-β treatment of IL-15 stimulated NK cells for 48 hours mimicked the results of the tumour cell-NK cell co-cultures by reducing the cell surface expression of NKp30, NKG2D and DNAM-1, but not NKp46 (Figure 2A). These changes were mirrored by reduced expression of the *NCR3* and *CD226* genes (encoding NKp30 and DNAM-1 respectively) but with little change in *NCR1* gene expression (encoding NKp46). Expression of the *KLRK1* gene (encoding NKG2D) was unaltered. However, cell surface expression of NKG2D requires association with its signalling chain, DAP10 [26], and expression of the *HCST* gene (encoding DAP10) was reduced in the presence of TGF-β. In contrast, TGF-β did not alter expression of the *CD247* gene (Figure 2B); this encodes CD3ζ, the signalling chain associated with NKp30 and NKp46. Comparing receptor expression (at the mRNA and protein level) in unstimulated NK cells with that in IL-15 stimulated, or IL-15 plus TGF-β treated NK cells, revealed that TGF-β exerted these inhibitory effects by antagonising IL-15 induced gene expression.

**Figure 3:**
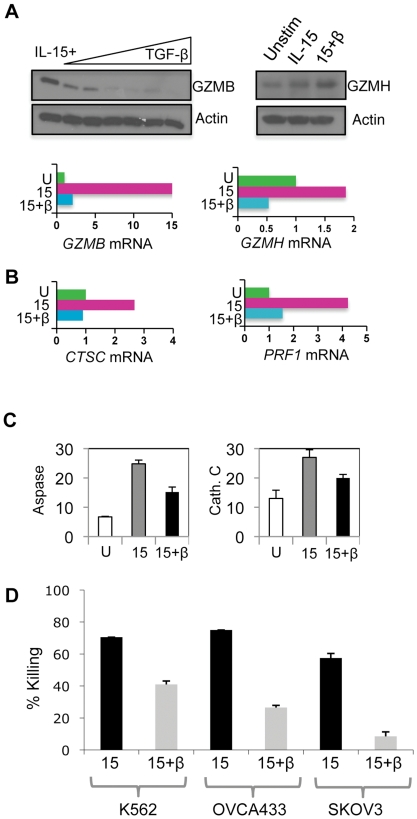
TGF-β inhibits expression of components of the NK cell cytotoxic apparatus. (A) Expression of granzymes B and H at the protein and mRNA level. For granzyme B, NK cells were stimulated 20 ng/ml IL-15 for 48 hrs and increasing amounts of TGF-β (ranging from 0 to 5 ng/ml). The immunoblot shows granzyme B expression (GZMB) and actin as a loading control. For granzyme H, expression was determined in unstimulated NK cells, NK cells treated with IL-15 and IL-15 plus TGF-β (for 48 hrs). The blots show granzyme H (GZMH) and actin levels. For gene expression, *GZMB* and *GZMH* steady state mRNA levels were quantitated from unstimulated (U), IL-15 stimulated (15) and IL-15 plus TGF-β (15+β) treated NK cells (using qRT-PCR) and expressed as arbitrary units, with expression in unstimulated NK cells defined as 1 unit (note the different scales). (B) Expression of the cathepsin C (*CTSC*) and perforin (*PRF1*) gene in unstimulated (U), IL-15 stimulated (15) and IL-15 plus TGF-β (15+β) treated NK cells, determined as in (A). (C) Protease activity in cytokine treated NK cells. Aspartase (Aspase) activity (a measure of granzyme B activity) assayed by hydrolysis of AcIEPD-pNA (left panel) and cathepsin C activity assayed by hydrolysis of GF-pNA (right panel). Activity was measured in lysates derived from unstimulated NK cells (U), NK cells stimulated for 48 hrs with 20 ng/ml IL-15 (15) or IL-15 plus 5 ng/ml TGF-β (15+β). Each reaction contained lysate from 6×10^5^ cells and was performed in triplicate, with the mean and SD calculated. Activity is expressed as arbitrary units. (D) NK cells were stimulated with either 20 ng/ml IL-15 (15) or IL-15 plus 5 ng/ml TGF-β (15+β) for 48 hrs and used in standard killing assays against K562, OVCA433 and SKOV3 tumour cell lines at an E∶T ratio of 3∶1 (with standard deviation shown).

Inhibition was not confined to alterations in NK cell surface receptors. As with mouse CD8^+^ T cells [24], TGF-β inhibited expression of multiple components of the NK cell cytotoxic apparatus at the mRNA and protein level. The fifteen-fold induction of *GZMB* gene expression resulting from IL-15 stimulation was antagonized by TGF-β treatment, whereas expression of the adjacent *GZMH* gene was much less responsive to these cytokines. These effects were manifested at the protein level (Figure 3A). Furthermore, expression of the perforin gene (*PRF1*) and *CTSC* (encoding the granzyme activating enzyme cathepsin C) were induced by IL-15 and antagonized by TGF-β (Figure 3B). The reduced expression of *CTSC* and *GZMB* was associated with reduced proteolytic activity associated with these molecules (Figure 3C). The cumulative effect of inhibiting NK cell activation receptor expression and components of the cytotoxic apparatus was reduced NK cell mediated killing of tumour target cells (Figure 3D). Thus, TGF-β treatment inhibited the IL-15 induced expression of key activating receptors and cytotoxic components involved in the detection and destruction of tumour cells.

### TGF-β allows IL-15 mediated NK cell survival

**Figure 4:**
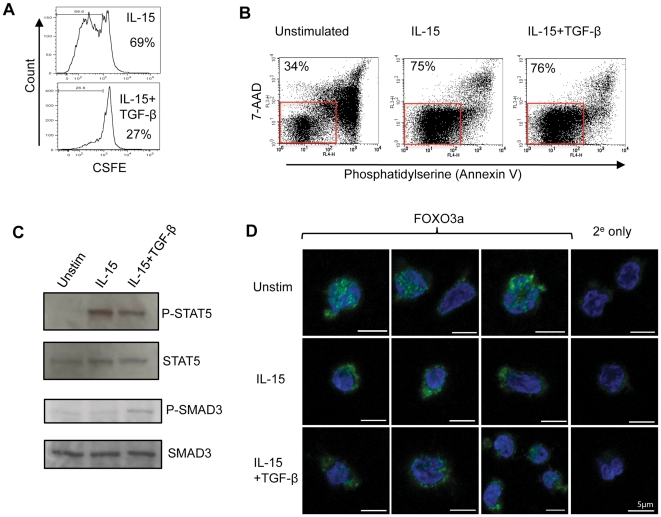
TGF-β does not inhibit IL-15 mediated NK cell survival. (A) NK cell proliferation *in vitro*. NK cells were labeled with CFSE and cultured for five days in IL-15 alone (top panel) or IL-15 plus TGF-β (bottom panel). Cell division (as assessed by reduction in CFSE staining) was determined by flow cytometry. The percentage of cells judged to have undergone at least one cell division is shown. (B) NK cell survival in the presence or absence of IL-15. NK cells were cultured for five days in the absence of exogenous cytokines (unstimulated) or in the presence of IL-15 or IL-15 plus TGF-β. Cell survival was assessed by staining with annexin V and 7-AAD. Healthy cells (lower left quadrant) are boxed and the percentage of cells within this box are shown. (C) Signalling pathways in NK cells. Immunoblots show the phosphorylation of STAT5 (P-STAT5) and SMAD3 (P-SMAD3) in unstimulated NK cells, IL-15 stimulated or IL-15 plus TGF-β treated. Total STAT5 and SMAD3 levels are shown as loading controls. (D) Intracellular localisation of the FOXO3 transcription factor in unstimulated NK cells, NK cells stimulated with IL-15 or NK cells treated with IL-15 plus TGF-β (48 hrs) analysed by confocal microscopy. The panels show FOXO3 (in green) and the nucleus (blue) with a scale bar of 5 µm. Also shown is staining in which the anti-FOXO3 antibody was omitted and cells were stained with a secondary antibody only (2^e^ only).

IL-15 also regulates NK cell survival [27], [28], [29]. The ability of TGF-β to inhibit IL-15 mediated events suggested that this survival might be impaired in the presence of TGF-β, as has been observed for cytotoxic T cells [30]. Human NK cell survival *in vitro* was enhanced by exogenous IL-15 but, unlike NK cell activation and proliferation, survival was not subject to inhibition by TGF-β (Figure 4A and B). TGF-β induced SMAD3 phosphorylation but did not substantially reduce IL-15 receptor proximal signalling events, as shown by its inability to suppress STAT5 phosphorylation (Figure 4C). In mouse NK cells, IL-15 mediated survival has been linked to the inhibition of FOXO3-dependent gene expression [29]. The phosphorylation of FOXO3 (via the PI3K/AKT pathway) causes its retention in the cytoplasm where it is unable to induce expression of pro-apoptotic genes [31], [32]. Human NK cells cultured in the absence of exogenous cytokines showed increased accumulation of FOXO3 in the nucleus. Stimulation with IL-15 redistributed FOXO3 from the nucleus to the cytoplasm, facilitating NK cell survival; this IL-15 induced redistribution of FOXO3 was unaffected by TGF-β treatment (Figure 4D).

### Localised inhibition of NK cell function in the human tumour microenvironment and its reversal by TGF-β blockade

**Figure 5:**
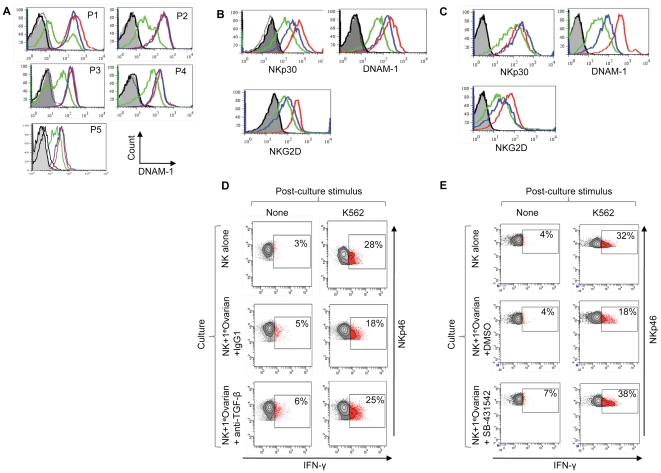
Human NK cell inhibition *in vivo* and restoration of effector function by TGF-β antagonists. (A) DNAM-1 expression on ovarian cancer patient-derived peripheral blood NK cells (blue histograms), NK cells from the patient's ascites (green histograms) and NK cells from peripheral blood of a healthy donor (red histograms). Samples were analysed without stimulation from five patients (P1-5). NKp30, NKG2D and NKp46 expression from these five patients is shown in Figure S3. (B and C) Restoration of NK cell activation receptor expression after TGF-β antagonism in NK cell-tumour co-cultures using either an anti-TGF-β antibody in (B) or SB-431542, a small molecule inhibitor of TGF-β family receptors in (C). Primary ovarian tumour cells (from ascites fluid) were co-cultured for 48 hrs with matched peripheral blood-derived NK cells (in the presence of IL-15). The tumour cell phenotype is shown in Figure S3B. Expression of NKp30, NKG2D and DNAM-1 was analysed on NK cells cultured in the absence of matched tumour (red histograms), in the presence of TGF-β antagonists (blue histograms) or control agents (green histograms). Antagonists were the anti-TGF-β antibody in (B) or SB-431542 (C), and control agents were a control antibody in (B) or DMSO, the solvent for SB-431542, in (C). Grey filled and black histograms are isotype controls. Experiments shown in panel (B) and (C) were performed using samples from different patients. (D) and (E) Restoration of NK cell effector function by TGF-β antagonism. Patient-derived tumour cells were co-cultured with autologous NK cells (from patient peripheral blood) and IL-15 for two days in the presence of TGF-β antagonist, or control agent, as indicated. Effector function was assessed by IFN-γ production after restimulation with K562. SB-431542 also restored effector function to NK cells co-cultured with HCT116 (Figure S4). Experiments shown in (D) and (E) were performed using samples from different patients.

Thus, despite antagonizing NK cell activation by IL-15, TGF-β does not counteract IL-15 mediated survival signals. This suggested that inhibited NK cells present in the tumour microenvironment might have their effector function restored if TGF-β activity could be interrupted. Obtaining human tumour-associated NK cells is challenging and functional studies of infiltrating NK cells are severely limited by the cell numbers available. However, the malignant ascites associated with ovarian cancer is a physiological niche from which tumour associated NK cells and autologous tumour cells can be relatively easily prepared and such tumour-associated NK cells are subject to localised inhibition [11]. We compared the cell surface phenotype of ascites-derived NK cells with that of matched NK cells from the patients' peripheral blood. As found previously [11], the cell surface expression of DNAM-1 was reduced on the tumour associated NK cells in all cases, whereas other activation receptors were variably affected (Figure 5A and Figure S3). In most cases, NK cells from the patients' peripheral blood were indistinguishable from those isolated from healthy donors, demonstrating localised inhibition *in vivo* and suggesting that NK cells entering the peritoneal tumour microenvironment (e.g. from the blood) become inhibited (Figure 5A and Figure S3). In agreement with this, it was possible to confer a tumour associated-like phenotype on the patient derived peripheral blood NK cells by co-culturing them with autologous tumour cells. This resulted in reduced cell surface expression of DNAM-1, NKG2D and NKp30 (Figure 5B and C), similar to the inhibition seen with tumour cell lines. These changes in receptor expression were dependent upon TGF-β, since an anti-TGF-β antibody partially restored expression of these molecules at the cell surface and boosted NK cell function accordingly (Figure 5B, D). Moreover, SB-431542, a small molecule inhibitor of the TGF-β signalling pathway [33], was capable of minimising the inhibition of autologous NK cells by tumour cells (Figure 5C, E). This inhibitor also partially restored the cell surface expression of activation receptors but, more remarkably, it enhanced IFN-γ synthesis to the levels produced by NK cells cultured in the absence of autologous tumour (Figure 5C and E). These effects were also observed when SB-431542 was used in co-cultures between NK cells and the colorectal cell line HCT116 (Figure S4). These results demonstrated that NK cell effector function could be efficiently restored, despite chronic interaction with autologous tumour cells.

## Discussion

These results establish a key role for TGF-β in the localised inhibition of human tumour-associated NK cells and reveal that this evasion pathway can be reversed by TGF-β antagonism. Knowledge of the role of TGF-β in tumour immune evasion comes largely from studies using animal models where disruption of TGF-β signalling, either by genetic means or using inhibitory agents, can restore anti-tumour immunity, identifying this evasion pathway as a promising therapeutic target [14], [20], [34]. In humans, circulating TGF-β levels correlate with poor prognosis in several cancer types and this has been linked with reduced NK cell activity and, in particular, with reduced expression of NKG2D on NK cells [16], [18], [35].

We have shown that chronic interactions occurring between human tumours and NK cells results in localised TGF-β mediated NK cell inhibition. Tumours fully exploit the activity of TGF-β; they produce it in large amounts, promoting angiogenesis and immunosuppression in the tumour microenvironment. The tumours themselves become refractory to the potent anti-proliferative activity of TGF-β via genetic instability and loss of responsiveness. Thus, whilst TGF-β suppresses the malignant phenotype in its early stages (e.g. via the inhibition of proliferation) it ultimately fosters an environment favoring tumour progression [14], [34]. In immune evasion, TGF-β exerts suppressive effects directly on effector cells (including cytotoxic cells) and indirectly by promoting the differentiation of regulatory T cells, which themselves inhibit cytotoxic cells [14], [36].

Inhibition of cytotoxic cells by TGF-β occurs via SMAD-dependent pathways and this targets the cytotoxic apparatus of both T cells and NK cells [22], [24]. In addition, SMAD3-dependent pathways suppress NK cell activation receptor expression [22]. TGF-β mediated inhibition of expression of DNAM-1, NKG2D and NKp30 was seen in all of the NK cell-tumour co-cultures performed. These receptors have an established role in tumour-mediated NK cell activation and their inhibition by TGF-β was associated with reduced granule exocytosis, tumour killing and IFN-γ release; effector pathways that are essential for robust anti-tumour immunity [1], [37], [38]. Bypassing activating receptors by stimulating the tumour-inhibited NK cells with PMA/ionomycin revealed that downstream signalling pathways remained active, suggesting that reduced cell surface expression of these receptors is key to human tumour immune evasion. In this regard, mice lacking these receptors are deficient in tumour immunity [39], [40], [41]. Indeed, these molecules are the targets of evasion strategies exerted by both tumours and viruses (such as Human Cytomegalovirus), indicating their central importance to NK cell function *in vivo* [42]. In ovarian cancer, DNAM-1 expression is also reduced on tumour associated NK cells as a result of receptor-ligand interactions [11]. Our results showed that chronic interactions between tumour cells and NK cells *in vitro* lead to the reduced expression of NKp30, NKG2D and DNAM-1, whereas only DNAM-1 was consistently affected *in vivo*. This difference is likely to reflect the multitude of evasion pathways that operate *in vivo* [6], [36], [43], [44], [45]. Interestingly, the TGF-β mediated inhibition observed here during chronic interactions required direct contact of NK cells and tumour cells, similar to that observed for NK cell inhibition mediated by myeloid suppressor cells [45].

Inhibition of the cytotoxic apparatus identifies a common mechanism by which TGF-β acts on NK cells and cytotoxic T cells. However, TGF-β appears to differentially regulate the survival of these cell types. TGF-β promotes the apoptosis of CD8^+^ T cells during the clonal expansions and contractions that occur during and after infection [30]. In contrast, our results show that whilst TGF-β inhibited IL-15 mediated proliferation and activation of NK cells, it did not inhibit IL-15 mediated NK cell survival. Both SMAD3 and STAT5 signalling pathways were active in cells treated with IL-15 plus TGF-β and the IL-15 dependent exclusion of FOXO3 from the nucleus was also unaffected by TGF-β treatment. In addition, both IL-15 and TGF-β signalling are coupled to AKT, the kinase responsible for FOXO3 phosphorylation [29], [46]. The mechanistic basis for the differential survival responses of cytotoxic T cells and NK cells remains unclear. However, it is likely to reflect fundamental requirements for the homeostatic control of NK and T cell responses to infection. Apoptosis of clonally expanding and contracting T cells is desirable to limit self-reactivity and to provide immunological space for repertoires of naïve cells. In contrast, NK cells are not antigen-specific and, whilst limited clonal expansions of NK cells do occur, there is not such a pressing need to eliminate expanded cells post-infection. Thus, in infection TGF-β blunts the effector function of activated NK cells but they presumably recover from this localised inhibition allowing them to respond to subsequent immunological challenge.

Although TGF-β may promote the apoptosis of tumour antigen-specific CD8^+^ T cells in the tumour microenvironment [30], our data shows that the NK cells remain viable, albeit in a relatively inactive state. Blocking TGF-β action may therefore allow recovery of NK cell effector function and anti-tumour immunity. We were able to reduce TGF-β mediated inhibition using an anti-TGF-β antibody and with a small molecule inhibitor (SB-431542) of TGF-β receptor signalling. Most importantly, these agents restored effector function to NK cells engaged in chronic interactions with autologous primary ovarian cancer cells, suggesting that TGF-β mediated immune evasion might be successfully blocked *in vivo*. Use of the anti-TGF-β antibody identifies the importance of TGF-β molecules themselves in the tumour-mediated evasion of NK cells. However, the SB-431542 molecule inhibits Activin Like Kinases (ALK)-4, -5 and -6 (33); these are components of several TGF-β family receptors and it is possible that this inhibitor is releasing NK cells from inhibition by other TGF-β family molecules in these assays; one such candidate is activin, which is known to inhibit NK cell production of IFN-γ [47].

A number of TGF-β antagonists are being evaluated as anti-cancer agents in clinical trials [14]. We have focused this study on the localised inhibition of human NK cells by tumour cells. However, within the tumour microenvironment the action of TGF-β is manifold and inhibition of TGF-β action could conceivably favor productive anti-tumour immunity via multiple pathways [14]. Whilst autoimmunity is a potentially damaging side effect of such treatment, successful elimination of tumours by the immune system does, by definition, require a degree of self-reactivity. The localised TGF-β mediated inhibition demonstrated here suggests that local delivery of TGF-β antagonists may help to reduce unwanted side effects arising from systemic blockade; ovarian cancer may respond to the local delivery of TGF-β antagonists used in conjunction with other immunotherapeutic agents, such as humanized antibodies against tumour cell surface molecules or cancer vaccines [48], [49]. Our results, together with the compelling evidence from animal models, argue that TGF-β antagonists offer great therapeutic potential for restoring anti-tumour immunity and suggest that the effects of these agents on the human immune system warrants careful evaluation.

## Materials and Methods

### Ethics statement

Patients with advanced ovarian cancer were undergoing paracentesis procedures as part of routine treatment for disease at the St. James's Institute of Oncology, Leeds UK. Peripheral blood samples were also taken from some patients. All patients gave informed, written consent. Approval for this study was provided by the Leeds Teaching Hospitals NHS Trust Research Ethics Committee.

### Cell lines, antibodies and flow cytometry

Human cell lines HCT116 (colon), HaCaT (immortalised keratinocyte) and Hela (cervical cancer) were cultured in DMEM with 10% FCS. The ovarian cancer cell lines OVCA433 and SKOV3 and the erythroleukaemic line K562 were cultured in RPMI supplemented with 10% FCS. Antibodies were purchased with the following fluorochromes; Allocophycyanin (APC), Fluorescein isothiocyanate (FITC) and phycoerythrin (PE). The following antibodies were used for flow cytometry (with fluorochromes and clone listed); CD56 (APC; clone NCAM 6.2), CD3 (FITC; clone HIT3a), NKp30 (PE; clone p30-15), NKG2D (PE; clone 1D11), DNAM-1 (PE; clone DX11), NKp46 (APC; clone 9E2), IFN-γ (PE; clone 4S.B3) CD107a (FITC; clone H4A3), MUC1 (FITC; clone HPMV), HER2 (FITC; clone Neu 24.7), CD138 (PE; clone mi15) all from Becton Dickinson (Oxford, UK). NKp46 (PE; clone 9E2) was from Miltenyi Biotec, MUC16 (PE; ×75) was from Gene Tex Inc. and TGF-β (PE; 1D11) was from R&D systems. Data was collected using an FACS Calibur or LSRII flow cytometer (BD Biosciences) and analysed using FACS Diva or Cellquest Pro (both from BD Biosciences) or Flowjo software (Treestar).

### Isolation and culture of human NK cells from blood and ascites

For peripheral blood, 20–30 ml blood samples were used to purify NK cells using a negative selection NK cell isolation kit (Miltenyi Biotec). Purified NK cells were subsequently cultured in DMEM, 10% human AB serum (Seralabs), 5% fetal calf serum (FCS, Gibco) at a density of 10^6^ cells/ml with no additions or 20 ng/ml recombinant human IL-15 and/or TGF-β 5 ng/ml (both from R&D systems), or as indicated. For ascites fluid samples (250–500 ml), cells were pelleted by centrifugation (400 g, 10 mins), washed in PBS, resuspended in RPMI supplemented with 10% FCS and plated for 24–48 hrs. The adherent cell fraction is heavily enriched for tumour cells. The non-adherent fraction (removed by washing) containing the NK cells was then applied to a Ficoll gradient (as used in the preparation of peripheral blood mononuclear cells) and, after centrifugation, the mononuclear fraction of cells was isolated and stained for expression of NK cell markers.

### Tumour-NK Co-cultures

NK cells were co-cultured in 6-well flat bottom plates with tumour cell lines or ovarian tumour cells at an NK∶tumour cell ratio of 1∶3. This is opposite to the typical 3∶1 ratio used in killing assays and helps to minimize killing during the co-culture and mimic the situation *in vivo* where tumour cells heavily outnumber NK cells. Each well contained 0.5-1×10^6^ cells and was cultured (for 48 hrs or as otherwise indicated) in the presence of 20 ng/ml IL-15+/−10 µM SB-431542 (Tocris Bioscience) or 10 µg/ml anti-TGF-β antibody (clone 1D11) or IgG1 isotype control antibody. At the end of the co-culture period, cells were stained with the appropriate antibodies and NK cells identified either by the CD56^+^CD3^−^ or NKp46^+^ phenotype. For degranulation and IFN-γ assays, NK cells were removed from the co-culture by gentle washing (the NK cells are weakly adherent). For degranulation, cells were then restimulated with K562 for 4 hrs, in the presence of anti-CD107a antibody and GolgiSTOP (from BD Biosciences). Cells were then harvested, stained with anti-NKp46 antibody and washed before data collection. For the IFN-γ production assay, NK cells were treated as for the CD107a assay and were then fixed and permeabilised using Leucoperm reagents (AB Serotec) and subsequently stained for intracellular IFN-γ.

For killing assays after co-culture, NK cells were purified from the co-culture using a MoFlo cell sorter (Dako Cytomation). Co-cultured cells were stained with anti-CD138 antibody and 7-aminoactinomycin D (7-AAD), the CD138^neg^ 7-AAD^neg^ population consisted of >95% CD56^+^CD3^neg^ NK cells. Sorted cells were used in a standard 5 hr killing assay with Cell Tracker Green (Invitrogen) labeled targets. Dead or dying target cells were identified after propidium iodide staining [50].

### NK cell proliferation and survival

Human NK cells were purified as above and loaded with 10 µM Carboxyfluorescein succinimidyl ester-SE (CFDA-SE; Invitrogen) for 10 mins at 37°C. Subsequently, 1 ml of FCS was added and the cells were pelleted (300 g, 10 minutes at room temperature) and washed twice with media. CSFE-labeled cells were cultured with IL-15+/− TGF-β as above. At day 5 cells were assessed for proliferation by excitation at 488 nm using an LSRII Flow cytometer (BD Biosciences). Data was analysed using FlowJo software (Treestar). For survival assays, purified NK cells were cultured in NK media with no additions or supplemented with IL-15 or IL-15 plus TGF-β for 5 days. Cells were stained with 7-AAD and APC conjugated annexin V in 1× staining buffer (all from BD Biosciences) to reveal cell surface phosphatidylserine and healthy cells identified as the 7-AAD^neg^Annexin V^neg^.

### FOXO3 localization by confocal microscopy

Indirect immunofluorescence of primary NK cells was performed on unstimulated and stimulated NK cells with IL-15+/−TGF-β (for 48 hrs), as above. Cells were allowed to adhere to coverslips coated in poly-L-lysine (Sigma-Aldrich), fixed with 4% paraformaldehyde for 20 mins and permeabilized using 0.5% saponin (Sigma-Aldrich) for 10 mins before blocking in PBS containing 1% BSA (Sigma-Aldrich). Coverslips were incubated with rabbit polyclonal antibody against FOXO3a (Cell Signaling Technology) for 1 hr, at room temperature and washed three times in PBS followed, by an AlexaFluor488-conjugated goat anti-rabbit secondary antibody (from Molecular Probes) for 1 hr at room temperature. Following three PBS washes, coverslips were mounted onto slides using ProLong Gold with DAPI to reveal the nucleus (from Molecular Probes). Images were acquired with a Nikon Eclipse C1 inverted scanning confocal microscope equipped with an attached Hamamatsu digital camera and a ×100 Plan Apochromat 1.4 NA oil objective (Nikon Instruments). Figures were assembled and annotated using Adobe Photoshop 4.

### Expression analysis; mRNA, protein and enzymatic assays

Steady state mRNA levels were determined by quantitative reverse transcriptase (RT)-PCR in NK cell cultures (48 hrs, with indicated cytokines) using Taqman Express plates run on a 7900 HT Taqman system (ABI). Total RNA was isolated from 1-1.5×10^6^ NK cells using the GeneElute Mammalian Total RNA isolation reagent (Sigma-Aldrich), samples were DNase treated (Ambion) and 2 µg of RNA reverse transcribed using the high capacity RNA-to-cDNA kit (from ABI). Resultant cDNA was quantitated using TaqMan primers and probes from ABI as follows (gene, assay ID); *NCR1*, Hs00183118_m1; *KLRK1*, Hs00183683_m1; *NCR3*, Hs00394809_m1; *CD226*, Hs00170832_m1; *GZMB*, Hs00188051_m1; *GZMH*, Hs00277212_m1; *PRF1*, Hs00169473_m1; *HCST*, Hs00367159_m1; *CD247*, Hs00167901_m1. Gene expression was analysed using the comparative Ct method (using 18S rRNA as the control) and are representative of data from three separate donors, each with qRT-PCR reactions performed in triplicate. Data are presented as fold change relative to unstimulated NK cell samples (denoted as 1 arbitrary unit).

For protein expression, immunoblotting of granzyme B and granzyme H (and the actin control) were performed using lysates derived from 6×10^4^ or 1×10^6^ NK cells respectively as described previously (50). For phosphorylated STAT5 and SMAD3, 1×10^6^ NK cells were lysed in 20 µl of RIPA buffer supplemented with protease inhibitors (Set III without EDTA; Sigma Aldrich, Dorset, UK) and 1× PhosSTOP phosphatase inhibitors (Roche), cleared by centrifugation at 16000 g for 5 mins before loading onto a 10% SDS-PAGE gel. Proteins were subsequently transferred and membranes blocked with 5% BSA, 0.1% Tween 20 in TBS for 1 hr at room temperature. Blots were probed with primary antibody overnight at a dilution of 1∶1000, in TBS containing 2.5% BSA, 0.1% Tween 20, washed 4 times in TBS, 0.1% Tween 20, followed by horseradish peroxidase conjugated anti-Rabbit IgG antibody at 1∶2000, left for 1 hr at room temperature and washed as before. Antibodies used were phospho-SMAD3 (Ser423/425; clone C25A9) and reprobed for SMAD3 (C667H9); phospho-STAT5 (Tyr694) and reprobed for STAT5 (3H7) all from Cell Signaling. Proteins were visualized using ECL (GE Healthcare). STAT5 phosphorylation was analysed after 48 hrs of cytokine treatment whereas SMAD3 was analysed after 30 minutes.

For enzymatic assays, granzyme B activity was analysed as aspartase activity by hydrolysis of the substrate AcIEPD-pNA (Calbiochem) and cathepsin C activity by hydrolysis of GF-pNA (Sigma Aldrich) as previously described [50].

## Supporting Information

Figure S1 **Inhibition of human NK cells following chronic interaction with tumour cells.** (A) Expression of activation receptors (as indicated) by NK cells cultured in the presence of IL-15, either with (green histogram) or without (red histogram) the colorectal tumour cell line HCT116. Isotype control stains are shown in grey and black. Co-culture was performed for the time indicated and NK cells identified in the co-culture as NKp46+ cells. (B) NK cell production of IFN-γ following chronic interaction with HCT116 cells for 48 hours. NK cells were cultured with IL-15 in the presence or absence of HCT116 cells as indicated. The NK cells were separated from the tumour and restimulated with K562 cells or PMA/ionomycin (as indicated). The percentage of cells producing IFN-γ is indicated. (C) NK cell granule exocytosis following chronic interaction with HCT116 cells. This experiment was performed as in (*B*), except that granule exocytosis was assayed by cell surface expression of the granule membrane protein CD107a. In addition, NK cells were separated from the tumour and cultured with IL-15 alone to test recovery (as indicated). (D) Localised inhibition is reversible. Expression of activation receptors (as indicated) by NK cells cultured in 20 ng/ml of IL-15 in the presence (green histogram) or absence (red histogram) of the colorectal tumour cell line HCT116. NK cells were cultured in IL-15 alone or IL-15 plus HCT116 cells for nine days, or for two days after which NK cells were removed (by gentle washing) and cultured with IL-15 in the absence of HCT116 for a further seven days (blue histogram). (E) NK cell inhibition by HCT116 requires cell-cell contact. Expression of activation receptors (as indicated) was determined in 48 hr cultures of NK cell alone (in 20 ng/ml IL-15; red histogram) or where the NK cells were separated from the HCT116 in a transwell dish (green histogram). Black and grey are isotype controls. (F) Inhibition by other tumour types. Expression of activation receptors (as indicated) by NK cells cultured in 20 ng/ml of IL-15 in the presence (green histogram) or absence (red histogram) of the ovarian cancer cell lines SKOV3 or OVCA433 (as indicated) for 48 hrs. NK cells were identified in the co-culture by expression of NKp46. (G) NK cell production of interferon (IFN)-γ following chronic interaction with SKOV3 or OVCA433 cells for 48 hours. NK cells were cultured in the presence of 20 ng/ml IL-15 in the presence or absence of the indicated tumour cell line. NK cells were removed from the culture (by washing) and restimulated with the susceptible NK cell target K562 for 5 hrs at an effector to target (E∶T) ratio of 1∶1 and IFN-γ production assayed by intracellular staining. NK cells were identified by NKp46 expression. The percentage of cells producing IFN-γ is indicated. (H) Inhibition by tumour is greater than immortalised cells. Expression of activation receptors (as indicated) by NK cells cultured in 20 ng/ml of IL-15 alone (red histogram) in the presence of Human Papillomavirus transformed keratinocytes (the cervical carcinoma cell line HeLa; green histogram) or with immortalised keratinocytes (HaCat; blue histogram) for 48 hrs. (I) NK cell granule exocytosis following chronic interaction with tumour cells (HeLa) or immortalised keratinocytes (HaCat). This experiment was performed as in (*D*), except that granule exocytosis was assayed by cell surface expression of the granule membrane protein CD107a.
(TIF)

Figure S2 **TGF-β dependent inhibition of NK cell killing activity following chronic interactions.** NK cells were cultured for 48 hrs in 20 ng/ml IL-15 alone or in the presence of the tumour cell line, HCT116. NK cells cultured with HCT116 included either an anti-TGF-β antibody or an isotype control antibody. After 48 hrs, NK cells were sorted from all cultures. The HCT116 cells express CD138 and NK cells were sorted based on the absence of this marker. Sorted NK cells (>95% purity) were used in a flow cytometric based killing assay of K562 cell line at an E∶T ratio of 2∶1. The data shows the mean of three experiments performed in triplicate, with standard deviation. Probability (P) values are indicated (calculated using a Mann-Whitney test).
(TIF)

Figure S3 **Localised NK cell inhibition by human tumours** ***in vivo*** **.** (A) Expression of activation receptors (as indicated) on NK cells derived from the peripheral blood of healthy donors (red histograms), the peripheral blood of five ovarian cancer patients (P1–P5; blue histograms) and autologous tumour-associated NK cells from the ascites fluid of P1–P5 (green histograms). NKG2D expression was not analysed in sample P4. The grey and black histograms are isotype controls. (B) Ascites-derived tumour cells express epithelial markers associated with ovarian cancer. Ascites were plated and cells left to adhere (for 24–48 hrs). Non-adherent cells were used as a source of NK cells and the adherent cells were analysed for cell surface expression of HER2, MUC1 and MUC16 associated with the ovarian cancer phenotype [11] and the NK cell markers NKp46 and CD56. In addition, adherent cells were tested for intracellular expression of TGF-β. The adherent cell samples contained no more than 10% fibroblasts. (C) Relative proportions of CD56^bright^ and CD56^dim^ NK cells in the NK cell populations derived from patient blood and patient ascites (patients 1–4) as well as from healthy controls. NK cells were identified as CD56+CD3^neg^ and the percentage of CD56^bright^ NK cells are indicated on each plot. The results show that the tumour associated NK cells have a higher proportion of CD56^bright^ NK cells compared to the matched peripheral blood (and to blood from healthy controls), as previously shown (11). For Patient 5, NK cells were identified via NKp46 expression and CD56^bright^ and CD56^dim^ data was not analysed. For patients 1–4, we analysed the expression of DNAM-1 on the CD56^bright^ and CD56^dim^ NK cells in the matched ascites and blood samples. The figures in brackets correspond to the ratio of DNAM-1 expression (as assessed by the geometric mean of fluorescence) in ascites/peripheral blood NK cells for CD56^bright^ and CD56^dim^ cells. Thus for patient 1, the values (0.14, 0.12) indicate that DNAM-1 expression in ascites derived CD56^bright^ NK cells was 0.14 times that observed in peripheral blood and for CD56^dim^ NK cells, DNAM-1 was expressed at 0.12 times that in peripheral blood. This data indicates that DNAM-1 expression was downregulated at similar levels on both subsets of tumour-associated NK cells.
(TIF)

Figure S4 **Antagonism of TGF-β mediated inhibition following chronic interactions.** (A) Effect of the TGF-β signalling inhibitor SB-431542 on inhibition mediated by HCT116. NK cells were cultured in 20 ng/ml IL-15 alone or with HCT116, in the presence of either SB-431542 or DMSO (as indicated). NK cells were removed from the culture (by gentle washing) and restimulated with the NK cell target K562 for 5 hrs at an effector to target (E∶T) ratio of 1∶1 and IFN-γ production assayed by intracellular staining. NK cells were identified by NKp46 expression. The percentage of cells producing IFN-γ is indicated. (B) NK cell granule exocytosis following chronic interaction with HCT116 cells in the presence of SB-431542 or DMSO. This experiment was performed as in (*A*), except that granule exocytosis was assayed by cell surface expression of the granule membrane protein CD107a.
(TIF)
