# Supplementary figures and images for: Human Tumour Immune Evasion via TGF-β Blocks NK Cell Activation but Not Survival Allowing Therapeutic Restoration of Anti-Tumour Activity
Source: PLoS One. 2011 Sep 6;6(9):e22842. doi: 10.1371/journal.pone.0022842 (PMC3167809; doi:10.1371/journal.pone.0022842)

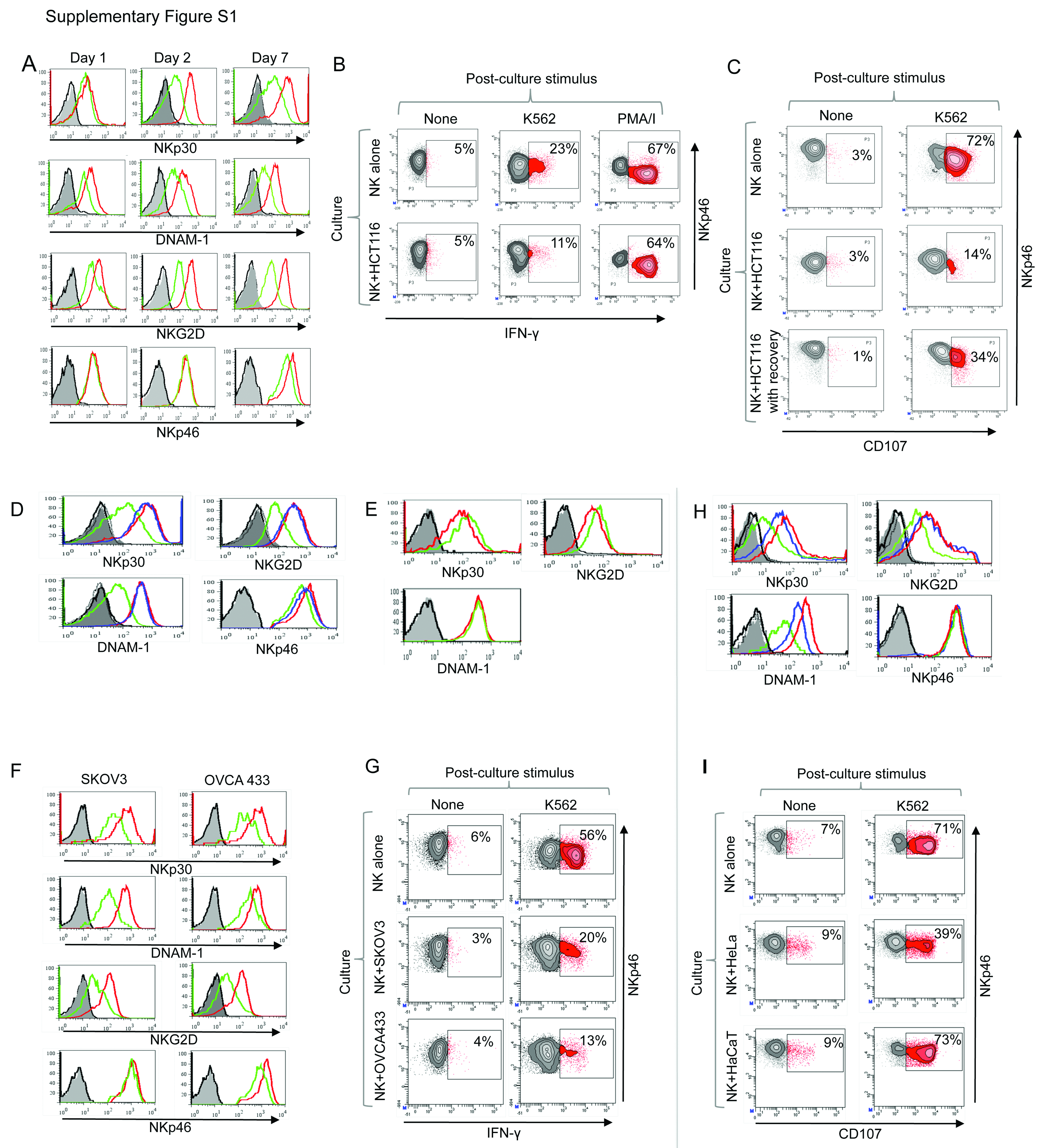

Supplement: Figure S1 — Inhibition of human NK cells following chronic interaction with tumour cells. (A) Expression of activation receptors (as indicated) by NK cells cultured in the presence of IL-15, either with (green histogram) or without (red histogram) the colorectal tumour cell line HCT116. Isotype control stains are shown in grey and black. Co-culture was performed for the time indicated and NK cells identified in the co-culture as NKp46+ cells. (B) NK cell production of IFN-γ following chronic interaction with HCT116 cells for 48 hours. NK cells were cultured with IL-15 in the presence or absence of HCT116 cells as indicated. The NK cells were separated from the tumour and restimulated with K562 cells or PMA/ionomycin (as indicated). The percentage of cells producing IFN-γ is indicated. (C) NK cell granule exocytosis following chronic interaction with HCT116 cells. This experiment was performed as in (B), except that granule exocytosis was assayed by cell surface expression of the granule membrane protein CD107a. In addition, NK cells were separated from the tumour and cultured with IL-15 alone to test recovery (as indicated). (D) Localised inhibition is reversible. Expression of activation receptors (as indicated) by NK cells cultured in 20 ng/ml of IL-15 in the presence (green histogram) or absence (red histogram) of the colorectal tumour cell line HCT116. NK cells were cultured in IL-15 alone or IL-15 plus HCT116 cells for nine days, or for two days after which NK cells were removed (by gentle washing) and cultured with IL-15 in the absence of HCT116 for a further seven days (blue histogram). (E) NK cell inhibition by HCT116 requires cell-cell contact. Expression of activation receptors (as indicated) was determined in 48 hr cultures of NK cell alone (in 20 ng/ml IL-15; red histogram) or where the NK cells were separated from the HCT116 in a transwell dish (green histogram). Black and grey are isotype controls. (F) Inhibition by other tumour types. Expression of activation rec [file pone.0022842.s001.tif]

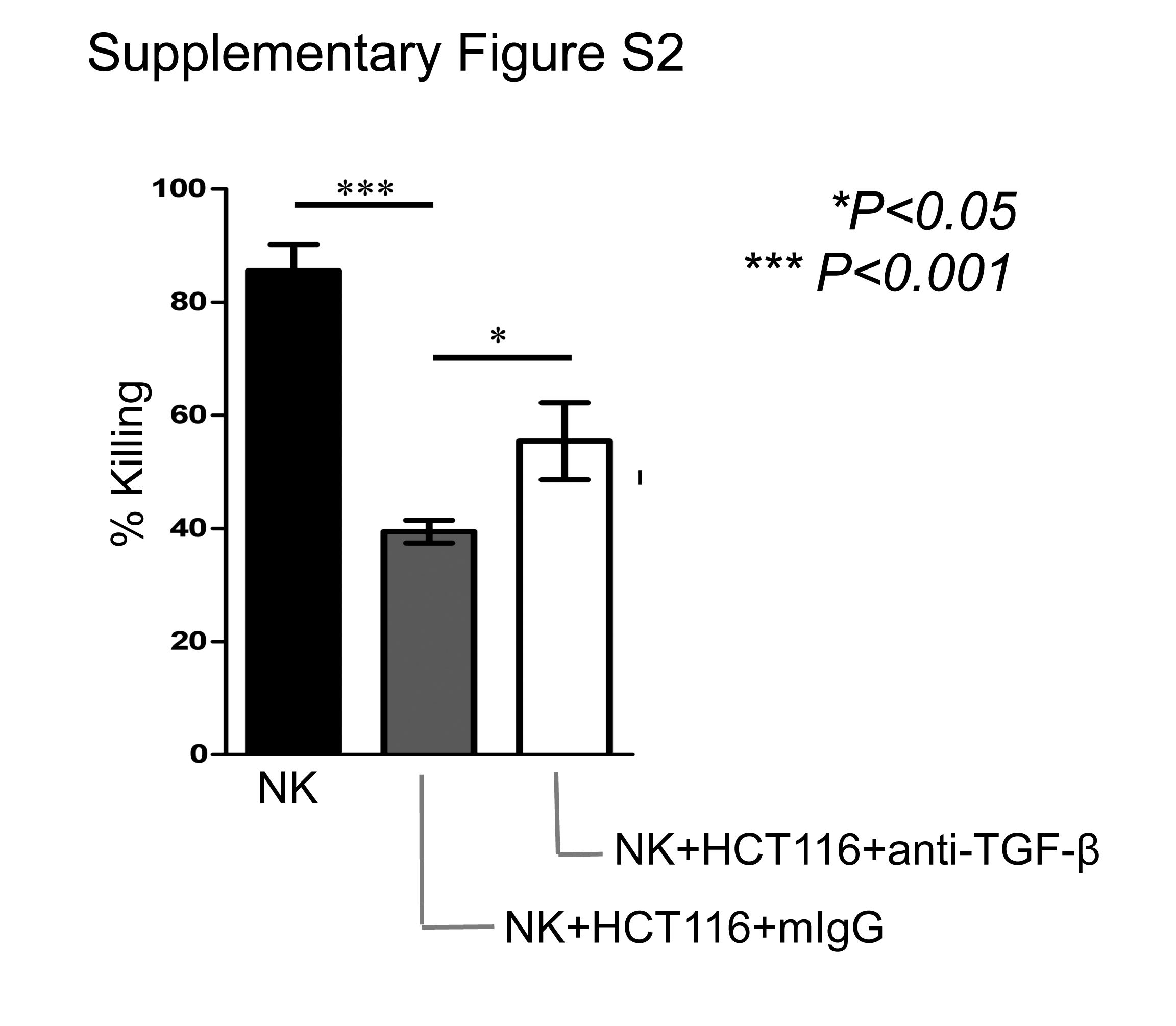

Supplement: Figure S2 — TGF-β dependent inhibition of NK cell killing activity following chronic interactions. NK cells were cultured for 48 hrs in 20 ng/ml IL-15 alone or in the presence of the tumour cell line, HCT116. NK cells cultured with HCT116 included either an anti-TGF-β antibody or an isotype control antibody. After 48 hrs, NK cells were sorted from all cultures. The HCT116 cells express CD138 and NK cells were sorted based on the absence of this marker. Sorted NK cells (>95% purity) were used in a flow cytometric based killing assay of K562 cell line at an E∶T ratio of 2∶1. The data shows the mean of three experiments performed in triplicate, with standard deviation. Probability (P) values are indicated (calculated using a Mann-Whitney test). (TIF) [file pone.0022842.s002.tif]

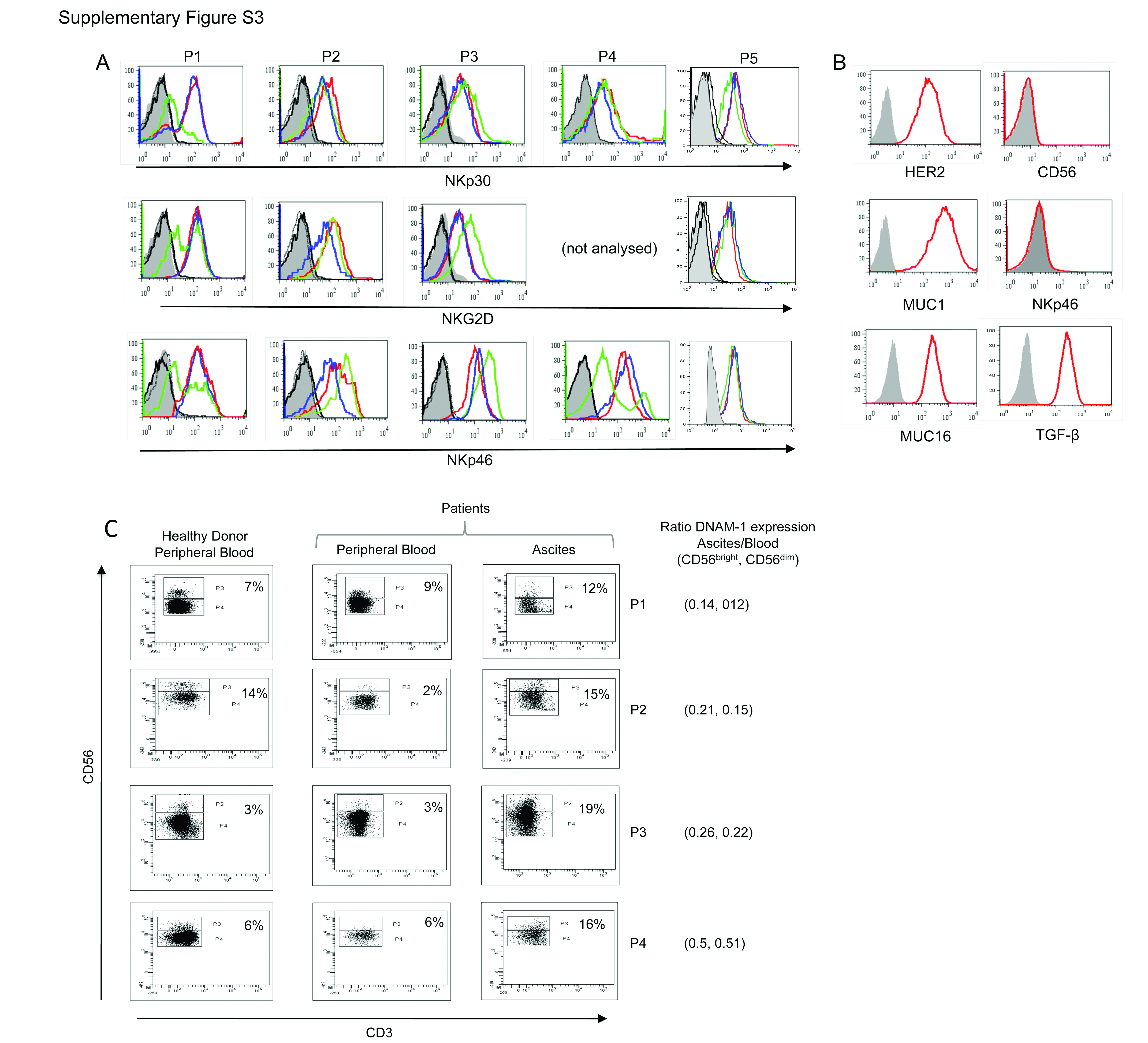

Supplement: Figure S3 — Localised NK cell inhibition by human tumours in vivo . (A) Expression of activation receptors (as indicated) on NK cells derived from the peripheral blood of healthy donors (red histograms), the peripheral blood of five ovarian cancer patients (P1–P5; blue histograms) and autologous tumour-associated NK cells from the ascites fluid of P1–P5 (green histograms). NKG2D expression was not analysed in sample P4. The grey and black histograms are isotype controls. (B) Ascites-derived tumour cells express epithelial markers associated with ovarian cancer. Ascites were plated and cells left to adhere (for 24–48 hrs). Non-adherent cells were used as a source of NK cells and the adherent cells were analysed for cell surface expression of HER2, MUC1 and MUC16 associated with the ovarian cancer phenotype [11] and the NK cell markers NKp46 and CD56. In addition, adherent cells were tested for intracellular expression of TGF-β. The adherent cell samples contained no more than 10% fibroblasts. (C) Relative proportions of CD56bright and CD56dim NK cells in the NK cell populations derived from patient blood and patient ascites (patients 1–4) as well as from healthy controls. NK cells were identified as CD56+CD3neg and the percentage of CD56bright NK cells are indicated on each plot. The results show that the tumour associated NK cells have a higher proportion of CD56bright NK cells compared to the matched peripheral blood (and to blood from healthy controls), as previously shown (11). For Patient 5, NK cells were identified via NKp46 expression and CD56bright and CD56dim data was not analysed. For patients 1–4, we analysed the expression of DNAM-1 on the CD56bright and CD56dim NK cells in the matched ascites and blood samples. The figures in brackets correspond to the ratio of DNAM-1 expression (as assessed by the geometric mean of fluorescence) in ascites/peripheral blood NK cells for CD56bright and CD56dim cells. Thus for patient 1, the values (0.14, 0.12) indicate that DNAM-1 ex [file pone.0022842.s003.tif]

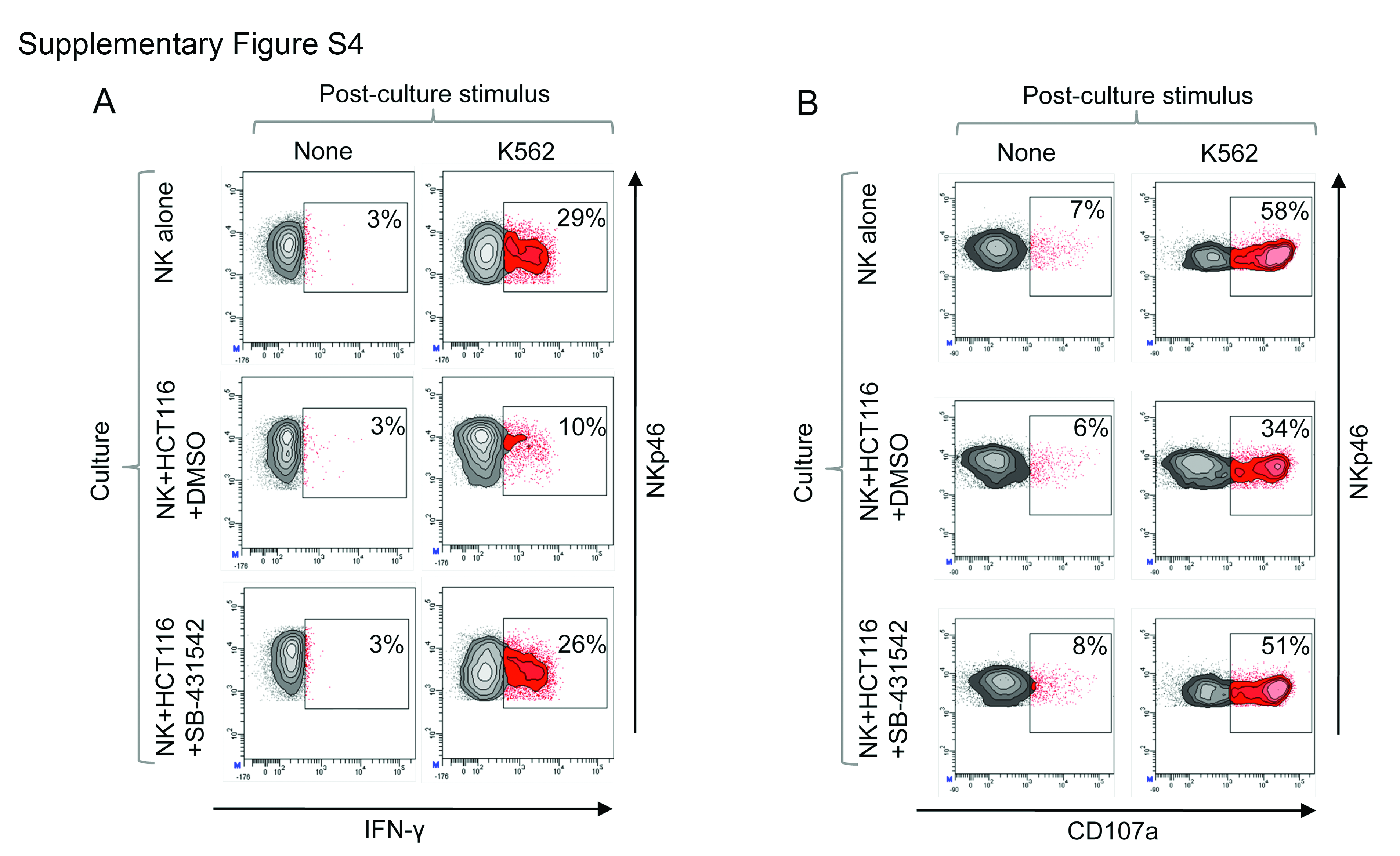

Supplement: Figure S4 — Antagonism of TGF-β mediated inhibition following chronic interactions. (A) Effect of the TGF-β signalling inhibitor SB-431542 on inhibition mediated by HCT116. NK cells were cultured in 20 ng/ml IL-15 alone or with HCT116, in the presence of either SB-431542 or DMSO (as indicated). NK cells were removed from the culture (by gentle washing) and restimulated with the NK cell target K562 for 5 hrs at an effector to target (E∶T) ratio of 1∶1 and IFN-γ production assayed by intracellular staining. NK cells were identified by NKp46 expression. The percentage of cells producing IFN-γ is indicated. (B) NK cell granule exocytosis following chronic interaction with HCT116 cells in the presence of SB-431542 or DMSO. This experiment was performed as in (A), except that granule exocytosis was assayed by cell surface expression of the granule membrane protein CD107a. (TIF) [file pone.0022842.s004.tif]
